# Male-Specific Association between a γ-Secretase Polymorphism and Premature Coronary Atherosclerosis

**DOI:** 10.1371/journal.pone.0003662

**Published:** 2008-11-06

**Authors:** Karen M. J. van Loo, Tim Dejaegere, Martine van Zweeden, Jessica E. van Schijndel, Cisca Wijmenga, Mieke D. Trip, Gerard J. M. Martens

**Affiliations:** 1 Department of Molecular Animal Physiology, Radboud University Nijmegen, Donders Institute for Brain, Cognition and Behaviour and Nijmegen Centre for Molecular Life Sciences (NCMLS), Nijmegen, The Netherlands; 2 Department of Molecular and Developmental Genetics, VIB, Leuven, Belgium; 3 Center for Human Genetics, KULeuven, Leuven, Belgium; 4 The Complex Genetics Section, Department of Biomedical Genetics, University Medical Center Utrecht, Utrecht, The Netherlands; 5 Department of Genetics, University Medical Center Groningen and University of Groningen, Groningen, The Netherlands; 6 Department of Cardiology, Academic Medical Center, Amsterdam, The Netherlands; Ohio State University Medical Center, United States of America

## Abstract

**Background:**

Atherosclerosis is a common multifactorial disease resulting from an interaction between susceptibility genes and environmental factors. The causative genes that contribute to atherosclerosis are elusive. Based on recent findings with a Wistar rat model, we speculated that the γ-secretase pathway may be associated with atherosclerosis.

**Methodology/Principal Findings:**

We have tested for association of premature coronary atherosclerosis with a non-synonymous single-nucleotide polymorphism (SNP) in the γ-secretase component APH1B (Phe217Leu; rs1047552), a SNP previously linked to Alzheimer's disease. Analysis of a Dutch Caucasian cohort (780 cases; 1414 controls) showed a higher prevalence of the risk allele in the patients (odds ratio (OR) = 1.35), albeit not statistically different from the control population. Intriguingly, after gender stratification, the difference was significant in males (OR = 1.63; p = 0.033), but not in females (OR = 0.50; p = 0.20). Since Phe217Leu-mutated APH1B showed reduced γ-secretase activity in mouse embryonic fibroblasts, the genetic variation is likely functional.

**Conclusion/Significance:**

We conclude that, in a male-specific manner, disturbed γ-secretase signalling may play a role in the susceptibility for premature coronary atherosclerosis.

## Introduction

Atherosclerosis is the basis of coronary artery disease and thought to be a multifactorial disease caused by susceptibility genes that act in concert with environmental factors. A number of susceptibility genes have been identified (e.g. apolipoprotein E (*APOE*) [1], low density lipoprotein receptor [2] and methylenetetrahydrofolate reductase [3]), but the signalling pathways responsible for vascular cell pathology are elusive. Interestingly, Wistar rats that display a high susceptibility for the dopamine receptor agonist apomorphine, the so-called apomorphine-susceptible (APO-SUS) rats [4], [5], show an impaired vasorelaxation to adrenergic stimuli when compared with their phenotypic counterparts APO-UNSUS rats [6], [7]. Impaired vasorelaxation is associated with an increased risk for the development of hypertension and vascular diseases such as atherosclerosis [8]. We recently identified a gene-dosage imbalance of the γ-secretase component Aph1b as the molecular-genetic basis of the difference between the APO-SUS and -UNSUS rats [9]. The γ-secretase enzyme complex cleaves many type I transmembrane proteins, including the amyloid-β (Aβ) precursor protein APP (known to be involved in neuronal amyloid plaque formation in Alzheimer's disease [AD]), NOTCH1-4, neuregulin, low-density lipoprotein receptor related protein (LRP1, 2 and 8) and N-cadherin [10], [11].

In view of the above findings, we hypothesize that the γ-secretase pathway may be linked to atherosclerosis. Increasing evidence suggests a link between altered vascular homeostasis, as seen in atherosclerosis, and the neurodegenerative disease AD. Apart from a partially overlapping epidemiology and an altered cholesterol homeostasis, atherosclerosis and AD have also been found to share genetic risk factors, including *APOE* and *LRP1* [1], [12]–[14]. Since the rare non-synonymous single-nucleotide polymorphism (SNP) Phe217Leu (rs1047552; T>G) in the human *APH1B* gene has recently been found to be associated with AD [15], we have now tested whether this SNP is also associated with premature coronary atherosclerosis.

## Results

### Male-specific association of *APH1B* Phe217Leu with premature coronary atherosclerosis

Since a gene-dosage imbalance of the *Aph1b* gene was the molecular-genetic basis of the APO-SUS/-UNSUS rat model [9] and the model was characterized by a disturbed endothelium-dependent vascular reactivity [6], [7], we tested the hypothesis that a genetic variation in the *APH1B* gene may contribute to atherosclerosis susceptibility in humans. In a Dutch case-control cohort consisting of 780 patients with premature coronary atherosclerosis and 1414 controls, we found a higher prevalence of the *APH1B* Phe217Leu risk allele (G-allele) in the patients, albeit not statistically different (χ^2^ = 2.09, *df* = 1, p = 0.15; OR = 1.35; CI = 0.90–2.01). Intriguingly, after gender stratification, the difference was significant in the male population (χ^2^ = 4.52, *df* = 1, p = 0.033; OR = 1.63; CI = 1.04–2.58), whereas females were not significantly different (χ^2^ = 1.62, *df* = 1, p = 0.20; OR = 0.50; CI = 0.17–1.48) (Table 1). Power analysis showed that for the detection in the female subpopulation of a risk effect similar to that observed in the male subpopulation, the power was insufficient (92% power for the total population; 81% power for the male subpopulation and 38% power for the female subpopulation, assuming a relative risk of 1.63, a disease allele frequency of 3.3% and a disease prevalence of 5%). All genotype distributions tested (cases and controls) fulfilled the Hardy-Weinberg criteria (data not shown).

**Table 1:** Genotype and allele frequencies for the *APH1B* Phe217Leu variation in a Dutch case-control study on premature coronary atherosclerosis.

	N	Genotype Frequencies (%)			Allele Frequencies (%)		*p*	OR
		TT	TG	GG	T	G		
**total**								
controls	1414	96.0	4.0	0	98.0	2.0		
patients	780	94.6	5.4	0	97.3	2.7	0.15	1.35
**males**								
controls	938	95.9	4.1	0	98.0	2.0		
patients	582	93.5	6.5	0	96.7	3.3	0.033^*^	1.63
**females**								
controls	476	96.0	4.0	0	98.0	2.0		
patients	198	98.0	2.0	0	99.0	1.0	0.20	0.50

Standard Chi-square tests were applied to evaluate the association with premature coronary atherosclerosis. OR = odds ratio; *p<0.05.

### Association of *APH1B* Leu217 with fibrinogen levels in premature coronary atherosclerosis

We then compared the association of the *APH1B* Leu217 allele with clinical parameters in the atherosclerosis patients, including the presence of risk factors (e.g. smoking behavior and occurrence of hypertension and diabetes mellitus), and the blood levels of lipid compounds (e.g. cholesterol, triglycerides, and low- and high-density lipoprotein cholesterol) (for a detailed overview of the parameters tested, see Table 2). These parameters were not related to the *APH1B* Phe217Leu variation (Table 2), except for a significant association (p = 0.028) with the fibrinogen levels in patients containing or lacking the *APH1B* Leu217 allele. Patients without the Leu217 allele displayed fibrinogen levels of 322.8±80.55 gr/l (n = 327, plus SD), whereas patients with the Leu217 allele had levels of 375.0±82.08 gr/l (n = 12, plus SD) (Table 2); due to low female patient numbers, gender stratification for fibrinogen levels was not possible. After Bonferroni adjustment for multiple comparisons, however, no statistically significant association of the fibrinogen levels with the Leu217 allele was detected (Bonferroni's adjustment requires a significance level of p≤0.00156).

**Table 2:** Clinical and biochemical characteristics of the premature coronary atherosclerosis patients with and without the *APH1B* 217Leu allele.

	without *APH1B* Leu217		with *APH1B* Leu217		*p*
	values±SD	n	values±SD	n	
Age (years)	43.3±5.3	739	42.9±5.3	42	0.60
BMI (kg/m2)	26.9±4.2	739	26.8±3.1	42	0.88
Age first manisfestation of vascular event (years)	41.6±5.9	739	40.9±6.1	42	0.47
Systolic blood pressure (mmHg)	129.1±17.2	739	127.0±18.5	42	0.45
Diastolic blood pressure (mmHg)	79.5±10.5	739	81.2±12.6	42	0.32
Total cholesterol prior to medication (mmol/l)	6.3±1.9	286	6.6±1.4	20	0.50
LDL-cholesterol prior to medication (mmol/l)	3.9±1.3	220	4.4±1.3	14	0.20
HDL-cholesterol prior to medication (mmol/l)	1.1±0.4	231	1.0±0.2	15	0.43
Triglycerids prior to medication (mmol/l)	1.8±1.2	229	1.9±0.7	15	0.77
Total cholesterol with medication (mmol/l)	5.0±1.5	738	4.9±1.7	42	0.88
LDL-cholesterol with medication (mmol/l)	3.0±1.2	730	3.1±1.8	41	0.78
HDL-cholesterol with medication (mmol/l)	1.1±0.3	737	1.1±0.4	42	0.41
Triglycerids with medication (mmol/l)	2.1±4.0	738	1.8±1.6	42	0.68
Apo A1 lipoprotein (mmol/l)	1.3±0.3	495	1.2±0.2	29	0.12
Apo B100 lipoprotein (mmol/l)	1.1±0.6	494	1.0±0.4	29	0.40
Lipoprotein (a) (mg/l)	215.2±309.4	496	240.7±377.1	30	0.66
Blood sedimentation rate (BSE) (mm/h)	10.4±11.3	345	9.4±5.2	16	0.74
Fibrinogen (gr/l)	322.8±80.5	327	375.0±82.1	12	0.028 *

	%	n	%	n	*p*
Acute myocardial infarction	73.34	739	69.05	42	0.54
Coronary angiography abnormal	98.23	621	97.14	35	0.64
Percutaneous transluminal coronary angioplasty	66.98	739	80.95	42	0.06
Coronary artery bypass grafting	7.98	739	4.76	42	0.45
Complaints claudication (<500 m walking distance)	11.77	739	7.14	42	0.36
Percutaneous transluminal angioplasty	7.58	739	7.14	42	0.92
Peripheral artery bypass grafting	2.84	739	0	42	0.27
Cerebro vascular accident	2.98	739	4.88	41	0.50
Smoking before the first event date	75.24	739	66.67	42	0.21
Treated for hypertension	25.44	735	33.33	42	0.26
Treated for diabetes mellitus	7.44	739	4.76	42	0.52
Hypercholesterolemia treatment first degree relative	48.37	552	48.57	35	0.98
Hypertension treatment first degree relative	45.71	617	47.37	38	0.84
Diabetes treatment first degree relative	27.42	660	23.08	39	0.55

Values are given as mean levels±SD or as percentages. N, number of individuals tested; BMI, body mass index; LDL, low density lipoprotein; HDL, high density lipoprotein; Apo, apolipoprotein. *p<0.05.

### Evolutionary conservation of amino acid residue Phe217 within the APH1 family

**Figure 1:**
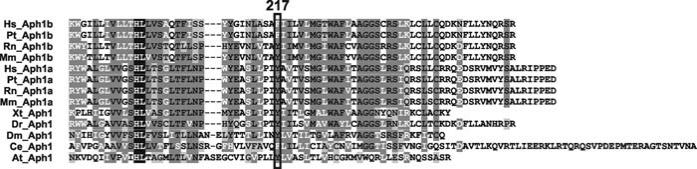
Alignment of vertebrate and invertebrate amino acid sequences of the region within APH1 surrounding residue 217. A black background indicates identical amino acid residues; a grey background indicates a conservative amino acid change. Abbreviations: Hs, *Homo sapiens*; Pt, *Pan troglodytes*; Rn, *Rattus norvegicus*; Mm, *Mus musculus*; Xt, *Xenopus tropicalis*; Dr, *Danio rerio*; Dm, *Drosophila melanogaster*; Ce, *Caenorhabditis elegans*; At, *Arabidopsis thaliana*. Sequences were aligned using Vector NTI (9.0) with default parameters.

The degree of conservation of an amino acid within a protein family is usually indicative of its importance for protein functioning. A multiple sequence alignment of members of the *APH1* family (Figure 1) showed that the Phe217 residue is conserved from plant, invertebrates, lower vertebrates, rodents and primates to man. The various APH1 proteins all contain at residue 217 either a phenylalanine (F) or the conservative change to tyrosine (Y). The Support Vector Machine (SVM) score (http://www.SNPs3D.org) [16] of −1.12 for Phe217Leu indicates a likely impact of this substitution on APH1B protein function.

### Functional analysis of the *APH1B* Phe217Leu polymorphism

**Figure 2:**
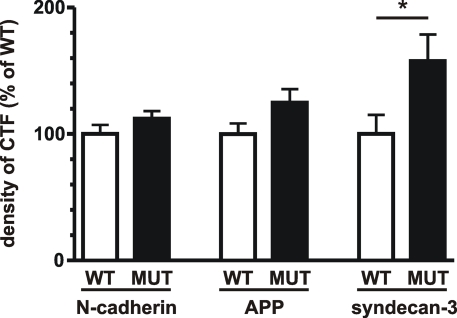
γ-Secretase cleavage activity of human wild-type APH1B (Phe217) and human mutant APH1B (Leu217) stably transfected into *Aph1abc^−/−^* mouse embryonic fibroblast cells. The levels of the C-terminal fragments (CTFs) of N-cadherin, APP and syndecan 3 were analysed in cells stably transfected with human APH1B Phe217 (WT) or human APH1B Leu217 (MUT). In the MUT cells, the CTF levels of N-cadherin and APP were slightly increased (1.1-fold and 1.2-fold, respectively), but the difference failed to reach significance. The levels of syndecan-3-CTF were significantly increased (1.6-fold; p = 0.044), indicating a reduced γ-secretase cleavage activity. Bars represent quantifications of CTF signals normalized to full-length protein levels in eight stably transfected cell lines with the average level in WT cell lines set to 100%. *: *p*<0.05.

We wondered whether the presence of a leucine instead of the conserved residue Phe217 of the APH1B protein would be of functional importance. *Aph1abc^−/−^* mouse embryonic fibroblasts were stably transfected with human APH1B Phe217 or Leu217. γ-Secretase activity was measured by quantifying the levels of different γ-secretase substrates in cell culture extracts. We observed a 1.6-fold reduction (p<0.05, n = 8) of γ-secretase activity towards one of its substrates, syndecan-3 [17], indicating a subtle influence on γ-secretase cleavage activity. The cleavages of two other substrates, N-cadherin and APP, were slightly but not significantly changed (Figure 2). Thus, in a substrate-dependent manner the Phe217Leu substitution affected γ-secretase cleavage activity.

## Discussion

In this study we show that the non-synonymous Phe217Leu polymorphism in the human *APH1B* gene is a male-specific risk factor for premature coronary atherosclerosis. The reduced γ-secretase cleavage activity of Leu217 APH1B, albeit in a substrate-specific manner, suggests a functional relevance of this polymorphism. Functional consequences of reduced gene dosage of *Aph1b* were also observed in the APO-SUS/-UNSUS rat model [9], an animal model with complex features, including an impaired vasorelaxation response to adrenergic stimuli [6], [7], which increases the risk for the development of hypertension and vascular diseases. The gene-dosage imbalance of the *Aph1b* gene (three gene copies in APO-UNSUS rats and one or two gene copies in APO-SUS rats) segregated with differences in γ-secretase cleavage activity and a number of other phenotypic characteristics [9]. We therefore speculate that a subtle effect on *APH1B* expression or function (copy number variation in rat or Phe217Leu variation in human, respectively) may affect γ-secretase activity and signalling downstream of gamma-secretase cleavage and, consequently, give rise to vascular complications. Interestingly, the *APH1B* Phe217Leu polymorphism was recently found to be also associated with AD [15]. AD pathogenesis is frequently characterized by a peripheral vascular disturbance and is often linked to other cholesterol/lipoprotein diseases, including atherosclerosis [18]. In addition, cerebral atherosclerosis has been found to be a strong contributory factor to sporadic AD pathogenesis [19]. The *APH1B* Phe217Leu variation may thus play a dual role by affecting atherosclerosis as well as AD pathogenesis, suggesting that the two diseases have converged and that the γ-secretase signalling pathway is a susceptibility pathway for vascular complications.

In line with the above supposition, a number of the γ-secretase substrates have been implicated in vascular pathogenesis. *LRP*, which belongs to a gene family involved in mediating cellular uptake of cholesterol-rich lipoproteins (the low-density lipoprotein receptor (*LDLR*) gene family), is highly expressed in atherosclerotic lesions [20], [21] and has been shown to represent a susceptibility gene for atherosclerosis [13]. The AD-associated γ-secretase substrate APP may be involved in vascular pathogenesis as well, since it metabolises cholesterol [22], physically interacts with LRP1 [23], and gives rise to the amyloid plaque constituents Aβ-40 and -42. Besides these well-studied substrates, the γ-secretase substrates NOTCH3, colony-stimulating factor 1 (CSF1), CD44, neuregulin and ERBB4 may also be involved in the vascular complications in patients with the *APH1B* Phe217Leu variation. Mutations in *NOTCH3* may cause cerebral autosomal dominant arteriopathy with subcortical infarcts and leukoecephelopathy (CADASIL), a syndrome characterized by systemic vascular smooth muscle cell (VSMC) degeneration [24]. CSF1 can contribute to atherosclerosis development via fatty streak formation and progression to complex fibrous lesions [25], and CD44 may enhance atherosclerosis pathogenesis via reactive oxygen species [26], whereas neuregulin and ERBB4 are necessary for vascular growth and development [27]–[29].

Our results show that, remarkably, only male individuals with atherosclerosis seem to be associated with the *APH1B* Phe217Leu variation. This might indicate a role for hormones or the involvement of a Y-chromosome-linked modulation. Such a gender-specific association has been observed for other SNPs as well, like a male-specific association of the *APOE2* and *APOE4* alleles with cardiovascular disease [30]. Thus, susceptibility to many common diseases may well be the result of complex interactions involving gender, genes and environmental factors. Following Bonferroni correction for multiple testing, no significant associations between the *APH1B* polymorphism and plasma lipoprotein parameters or other risk factors were observed in the atherosclerosis patients. More detailed studies will be necessary to establish the biochemical mechanisms underlying atherosclerosis in patients carrying the Phe217Leu polymorphism.

In the present study, association was only tested for premature coronary atherosclerosis, a disease with a substantial heritability [31]. To investigate whether the Phe217Leu SNP constitutes a susceptibility factor for other vascular complications, it would be of interest to also test for association in elderly atherosclerotic patients and in patients with other vascular defects (e.g. myocardial infarction). No significant association of the *APH1B* chromosomal region with coronary artery disease, myocardial infarction or other related diseases has been found in recent genome-wide association studies (GWAS) [32]–[35]. Thus, apart from the functional Phe217Leu polymorphism, the contribution of any additional genetic variant in the *APH1B* gene to the phenotype is expected to be small. Unfortunately, no results for the *APH1B* Phe217Leu polymorphism are available from the GWAS since this polymorphism was not represented on the arrays used in these studies. In addition, based on the data from the International HapMap-CEU project none of the SNPs tested in the GWAS is in near perfect proxy (r^2^≥0.8) to the Phe217Leu polymorphism. Therefore, the GWAS data do not provide any additional information concerning the functional *APH1B* Phe217Leu SNP.

In conclusion, our results suggest that the γ-secretase pathway is a candidate pathway for premature coronary atherosclerosis and warrant further studies on genetic variations in this pathway in various diseases with vascular complications.

## Materials and Methods

### Subjects

We selected consecutive Dutch premature coronary atherosclerosis patients (n = 780, age 43.3±5.3; 582 males, age 43.6±5.3 and 198 females, age 42.5±5.5) who qualified for inclusion after a myocardial infarction, surgical or percutaneous coronary revascularization, or a coronary angiogram with evidence of at least a 70% stenosis in a major epicardial artery (Atherosclerosis Outpatient Clinic of the Academic Medical Center of the University of Amsterdam). The study was approved by the Medical Ethical Committee of the Academic Medical Center (Amsterdam, The Netherlands) and all patients gave written informed consent. The control subjects (n = 1414, age 51.8±11.9; 938 males, age 53.7±10.9 and 476 females, age 48.2±12.9) were from the Sanquin Blood Bank. Volunteers were recruited at their blood donation session at one of the collection sites of the Sanquin Blood Bank covering the north west of the Netherlands. Therefore more than >95% of these donors lived in the Dutch postal code area 1000–4000. This area was chosen while the Amsterdam patient cohort geographically overlaps the region of the blood donor cohort serving as control donors.

### Biochemical analysis

In the atherosclerosis patients, plasma cholesterol and triglycerides were determined with commercially available enzymatic methods (Boehringer Mannheim, FRG, No. 237574, and Sera-PAK, No. 6639, respectively). To determine high-density lipoprotein cholesterol, the polyethylene glycol 6000 precipitation method was used. Low-density lipoprotein cholesterol was calculated by the Friedewald formula.

### APH1B genotyping

Following the isolation of genomic DNA, *APH1B* (MIM# 607630) Phe217Leu genotyping was performed via allele-specific PCR using primers specific for SNP rs1047552 (outer/general primers: forward: 5′-TGCCTTCTAGGGTTACCATCTGA-3′ and reverse: 5′-AGTCGGCTTTACACTGTCCCA-3′. Inner/specific primers: forward specific for the “T-allele”: 5′-AATAAACCTGGCGTCAGCATTT-3′ and reverse specific for the “G-allele”: 5′-GCCCATGAGCACCAGGATTATC-3′).

### Generation of Aph1abc^−/−^ mouse embryonic fibroblast (MEF) lines

Conditionally targeted (*Aph1a* and *Aph1c*) or classically targeted (*Aph1b*) mice were described before [36]. Animals carrying a null allele were obtained after breeding with transgenic mice expressing a *Pgk* driven Cre-recombinase. Mouse embryos were dissected at E8.5 from *Aph1abc^+/−^* crosses and genotype was determined by PCR analysis on yolk sacs. Mouse embryonic fibroblast (MEF) cultures were derived from dissociated *Aph1abc^−/−^* mouse embryos [37].

### Generation of stable cell lines

The Phe217 to Leu217 mutation was made using the QuikChange II site-directed mutagenesis kit (Stratagene), using human wild-type APH1B cloned into pcDNA3.1 Zeo+ (Invitrogen) as template. The primers used to introduce the mutation were: 5′-CCTGGCGTCAGCATTGATAATCCTGGTGCTC-3′ (forward); 5′-GAGCACCAGGATTATCAATGCTGACGCCAGG-3′ (reverse). Phe217 and Leu217 hAPH1B were recloned into pMSCVpuro* and cotransfected into HEK293 cells with helper plasmid pIK Ecopac for packaging into retroviruses. Retroviruses were harvested and snap frozen aliquots were stored at −80°C until use. MEF *Aph1abc^−/−^* cells were transduced with retrovirus for 24 hrs followed by puromycin selection in DMEM/F12 supplemented with 10% FCS until stable lines were obtained.

### Measurement of gamma-secretase activity towards different substrates

Stably transfected MEFs were seeded and grown to confluency. Cells were rinsed twice with ice-cold PBS and lysed in 1% Triton, and postnuclear fractions were isolated by centrifugation at 10,000 g for 15 min at 4°C. Proteins were quantified using a standard Bradford assay (Pierce) and 10–15 µg protein/lane was loaded on Bis-Tris SDS-PAGE gels (Invitrogen) and transferred to nitrocellulose membranes for Western blot detection for the indicated proteins. Gamma-secretase activity towards each substrate was expressed as the level of substrate C-terminal fragment (the direct gamma-secretase substrate) relative to levels of full-length protein. For densitometric quantification, the films were scanned using an Image Scanner (Amersham Pharmacia) and analyzed using ImageMaster.

### Antibodies

APP was detected with C-terminal pAb B63.1 and syndecan 3 with C-terminal mAb 2E9. An antibody against the N-cadherin C-terminus (clone 32, BD Bioscience) was purchased.

### Statistical analysis

Genotype frequencies were tested for the Hardy-Weinberg equilibrium. Differences between cases and controls were analysed by standard contingency table analysis using two-tailed χ^2^ test probabilities. Odds ratios (95% confidence intervals (CI)) were calculated as an index of the association of the *APH1B* genotypes with premature atherosclerosis. Continuous and categorical biochemical and clinical variables were determined with the Student's t-test and χ^2^-analysis, respectively. A p-value<0.05 was considered statistically significant (GraphPad Software Inc, San Diego, CA, USA). Though being conservative, Bonferonni correction was used to determine the significance of the biochemical and clinical variables. Power calculations were estimated using Quanto v1.2 [38].
